# Citizens' Attitudes, Knowledge, and Educational Needs in the Field of Omics Sciences: A Systematic Literature Review

**DOI:** 10.3389/fgene.2020.570649

**Published:** 2020-10-23

**Authors:** Giovanna Elisa Calabrò, Michele Sassano, Alessia Tognetto, Stefania Boccia

**Affiliations:** ^1^Section of Hygiene, University Department of Life Sciences and Public Health, Università Cattolica del Sacro Cuore, Rome, Italy; ^2^Department of Woman and Child Health and Public Health–Public Health Area, Fondazione Policlinico Universitario A. Gemelli Istituto di Ricovero e Cura a Carattere Scientifico (IRCCS), Rome, Italy

**Keywords:** citizens' literacy, omics sciences, genetic/omics knowledge, public attitudes, educational needs, systematic review, personalized medicine

## Abstract

**Background:** The huge development of omics sciences is changing the classical medical approach and making new technologies available. In this context, education of citizens is essential to allow appropriate decisions about their own health. Hence, we aimed to summarize existing literature regarding citizens' knowledge, attitudes, and educational needs on omics sciences.

**Methods:** We performed a systematic literature review (SLR) using Pubmed, ISI Web of Science, and Embase databases. The eligibility criteria for inclusion in this review required that the studies investigated knowledge, attitudes, or educational needs regarding omics sciences among the general population.

**Results:** We included 54 studies, published between 2006 and 2020. Most of the included studies (72%) investigated citizens' knowledge, half of them (56%) attitudes, and 20% educational needs in the field of omics sciences, while 52% investigated attitudes and perceptions about genetic and/or omics tests. Most studies (64%) reported a limited knowledge level among citizens, even though most (59%) reported participants understood the benefits of the use of omics sciences into medicine. As for omics tests, a controversial opinion toward their use into practice was reported among citizens. Most of the studies (82%) investigating citizens' educational needs highlighted a clear gap to be filled.

**Conclusions:** Our SLR summarizes current knowledge on citizens' literacy, attitudes, and educational needs on omics science, underlining the need for strengthening public engagement on this topic. Further research is needed, however, to identify appropriate methods and models to achieve such an improvement.

## Introduction

Rapid growth in genetic and genomic research has transformed our understanding of the role of genes in health and disease. The historical focus of genetic research has been on rare, single-gene disorders, in which disease risk is largely based on the presence or absence of a mutation in a single associated gene. This focus has been greatly expanded in recent years (Lea et al., 2011). Research in genomics is now examining the genetic components of common, complex diseases, such as cancer, diabetes, heart disease, etc. For these diseases, the contributions of single genes to risk are often small in comparison to the rare, inherited diseases, and disease risk is based on multiple genetic and environmental factors. These aspects highlighted the complexity of biological systems and provided new approaches to diseases diagnosis, treatment, and prevention (Lea et al., 2011).

Since the mapping of the human genome in 2003 (International Human Genome Sequencing Consortium, 2004), important progress has been made in understanding molecular and genetic pathways underpinning human health and diseases, promoting the development and the diffusion of genomics and of other omics sciences and related technologies.

In the last two decades, the use of words ending in “-omics” has extended, from the initial “genomics,” to a wide range of biomolecular disciplines addressed to the study of specific aspects considered as a whole. Therefore, the omics sciences study pools of biological molecules (e.g., ions, nucleic acids, proteins, enzymes) with various functions within living organisms and have the primary objective to analyze as a whole e.g., genes contained in DNA (genomics) and their multiple functions (functional genomics), DNA transcription product–RNA- (transcriptomics), proteins encoded by DNA through RNA (proteomics), molecules that interact within an organism or metabolites (metabolomics) (Lin and Qian, 2007; Tebani et al., 2016). Among the other goals of these sciences is also to study the connections and reciprocal interactions between the pool of biological molecules (interactomics) and between these molecules and microorganisms of the intestinal flora (microbiomics), foods and/or nutrients (nutribiomics) (Coughlin, 2014).

Since then, the Human Genome Project (HGP) was followed by other relevant initiatives, such as International HapMap Project (The International HapMap Consortium, 2003), ENCyclopedia of DNA Elements (ENCODE) Project (ENCODE Project Consortium, 2004), 1'000 Genome Project (1000 Genomes Project Consortium et al., 2015), and 100'000 Genome Project (Turnbull et al., 2018; Genomics England, 2019a,b). The importance of genetic factors in determining disease risk was understood by various European and not European governments and Institutions. To this end, a number of projects were put in place in the world, some of them even before HGP: for example, the Icelandic company deCODE, around 1996, announced a plan to genotype the entire national population (An, 2017).

Hence, an exponential growth of knowledge took place, first in genomics and later in other omics sciences too. As a consequence, new technologies, such as Next-Generation Sequencing (NGS), are nowadays available. These high-throughput omics technologies simultaneously measure thousands of data providing detailed information of cells, organisms, and populations and contributing to the definition of new diagnostic tests, new biomarkers and new drugs in the era of precision medicine (Tebani et al., 2016). These new second generation technologies led to an important cost reduction compared to the past (van Dijk et al., 2014), allowing then an increased accessibility to genomics testing and a potential improved sustainability by health systems. Therefore, during last years, a growing and uncontrolled availability of genetic tests, not only for monogenic disorders but also for multifactorial ones, took place and this progress in the genomics field had evident implications for public health, bringing important benefits but also potential risks for the population. A perfect example in this sense is given by direct-to-consumer genetic tests (DTC-GTs), which are tests sold by companies directly to consumers, without the involvement of a health professional (Su, 2013). In particular, besides potential discriminatory and privacy issues, not always properly addressed by laws and regulation (Kalokairinou et al., 2018; Hoxhaj et al., 2020), citizens don't have competencies needed to understand of results of these genetic tests, thus possibly leading to further unnecessary diagnostic investigation and, finally, waste of healthcare resources (Borry, 2010; Su, 2013). Furthermore, literature data suggest that knowing own genetic risk for specific diseases could lead to psychological distress, even though there are not clear evidence yet (Su, 2013).

All these aspects highlight the importance of counseling by trained health professionals and of appropriate citizens' education about omics sciences and new technologies related to them. Previous reviews report conflicting results about citizens' knowledge in the field of genetics, genomics, and DTC-GTs (Covolo et al., 2015; Hoxhaj et al., 2019; LePoire et al., 2019), even though with positive attitudes (Covolo et al., 2015; American Society of Human Genetics, 2020).

However, to our knowledge, no previous systematic review attempted to assess citizens' knowledge and attitudes in the wide field of omics sciences. Furthermore, we do not yet have adequate information on the educational needs of the general population in this field and on the topics of greatest interest to citizens.

Hence, to address these issues, our aim was to systematically summarize the existing evidence about knowledge, attitudes, and educational needs regarding omics sciences among the general population.

## Methods

### Search Strategy

An online search was conducted using the following electronic databases: Pubmed, ISI Web of Science and Embase. The search was limited to articles published in English language from January 1, 2003, until May 31, 2020. We used the following terms for the literature search in MEDLINE database: (“omics” OR “genomics” OR “omics sciences”) AND (“knowledge” OR “opinion” OR “perception” OR “awareness” OR “education” OR “literacy”) AND (“citizen^*^” OR “population” OR “public”). This search strategy was also used as template for the search in the other databases.

Three researchers (GEC, MS, AT) independently reviewed titles, abstracts, and full texts of the retrieved papers in order to identify the eligible studies. Results were cross-checked and any disagreement was resolved through discussion until consensus was reached. The snowball strategy, a manual search of the references reported by studies retrieved from the online databases, was also adopted to identify additional studies. The systematic review was drafted in accordance with PRISMA guidelines (Moher et al., 2009).

### Eligibility Criteria

Studies that investigated knowledge, attitudes (in terms of perceived benefits and/or risks), or educational needs regarding omics sciences among the general population were deemed as eligible. We excluded commentaries, editorials, conference abstracts, reviews, case reports, case series, and book chapters, and articles addressing a population of researchers or professionals only.

### Data Extraction

From each eligible study we extracted information on Country, promoter of the initiative, duration and aim of the initiative, topic, target population and age, methods/initiative description and initiative type, awareness on genetics/omics sciences, perceived benefits on the use of genetics/omics sciences in medicine, worries about the use of genetics/omics sciences in medicine, perceptions about genetic/omics tests, need for more education/information regarding omics sciences among the population.

Results are presented according to the three major areas investigated across the studies, namely knowledge, attitudes and educational needs about omics sciences and genetic and/or omics tests.

## Results

### Study Selection and Characteristics of Included Studies

Electronic databases search led to the identification of 1,948 articles (381 from Pubmed, 582 from ISI Web of Knowledge, and 985 from Embase). After duplicates removal and title and abstract screening, 99 articles were selected. From these, after full-text analysis 51 studies were removed, while 6 additional studies were retrieved though snowball search of reference lists of included articles.

**Figure 1:**
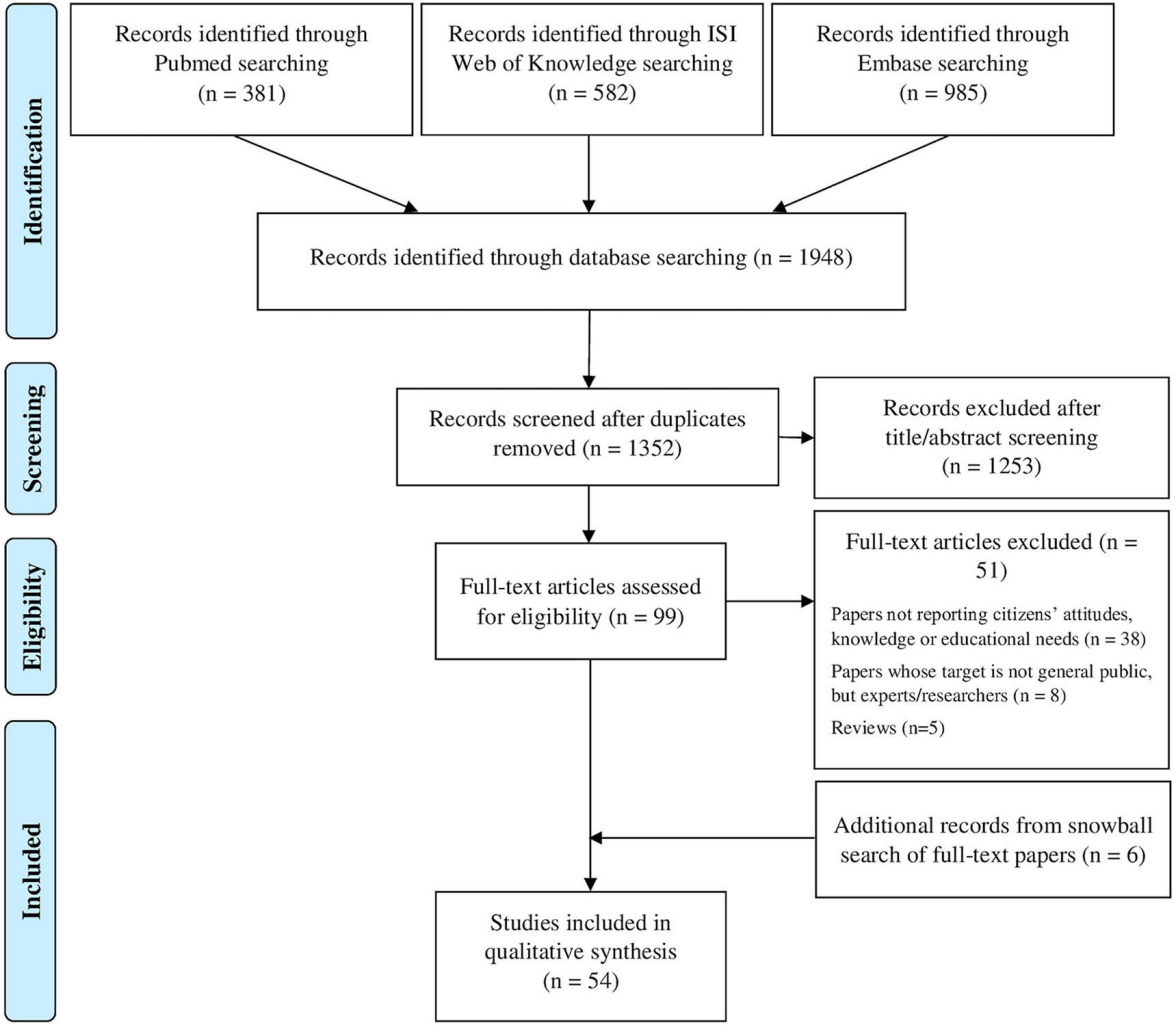
Study selection process flowchart.

After the latest selection process, 54 articles responded to eligibility criteria and were included in the systematic literature review. The Figure 1 shows study selection process, as indicated by PRISMA guidelines (Moher et al., 2009), while Table 1 reports main characteristics of included studies.

**Table 1:** Studies included in the systematic literature review and their main characteristics.

**References**	**Country**	**Initiative funder/promoter/supporter**	**Aims and methods**	**Target population**	**Age**
Fomous et al. (2006)	USA	University	Improvement of understanding about genomics and implications of the Human Genome Project on public health through an institutional portal called “Genetics Home Reference” (GHR) (http://ghr.nlm.nih.gov/)	General population	–
Henneman et al. (2006)	Netherlands	University	Assessment of attitudes toward availability and use of genetic tests through a questionnaire	General population (*N* = 817)	Males: mean: 57.6 years, range: 27–90 years Females: mean: 50.9 years, range: 25-93 years
Skirton et al. (2006)	UK	University	Assessment of the understanding of genetics and of attitudes toward genetic tests for clinical and research purposes, through 2 focus groups lasting 1.5–2 h each	Older adults (*N* = 7 e 10 in 2 focus groups)	≥65 years (68–90 years in group 1; 68–82 years in group 2)
Calsbeek et al. (2007)	Netherlands	Panel of Patients with Chronic Diseases is supported by ministerial funding	Assessment of genetic knowledge, attitudes toward genetic tests, and their relations and changes over time, through postal questionnaires administered in 2002 and 2004	Patients with asthma, diabetes mellitus and cardiovascular disease (1st survey−2002 *N* = 577; 2nd survey−2004 *N* = 398)	≥15 years
Goddard et al. (2007)	USA	Partially supported by Centers Disease Control and Prevention and by American Society of Human Genetics in Public Health Genomics Research and Practice	Assessment of awareness regarding nutrigenomics DTC tests through questionnaire	General population (*N* = 5250)	≥18 years
Morren et al. (2007)	Netherlands	University	Assessment of knowledge and attitudes regarding genetics and genetic tests through a postal questionnaire	Individuals with chronic diseases (*N* = 1496)	≥15 years
Ishiyama et al. (2008)	Japan	Supported by KAKENHI (research grant) by the Ministry of Education, Culture, Sports, Science, and Technology of Japan	Assessment of attitudes toward the promotion of genomics studies and relation between attitudes and the level of genomics literacy through a postal questionnaire	General population (*N* = 2171)	20–69 years
Goddard et al. (2009)	USA	State health departments, Behavioral Risk Factor Surveillance Systems (BRFSS)	Assessment of knowledge and use of nutrigenomics DTC tests through questionnaire	General population (Oregon *N* = 1867; Michigan *N* = 5499; Utah *N* = 2441; national *N* = 5250)	–
Makeeva et al. (2009)	Russia	Partly supported by Russian Foundation for Basic Research and SB RAMS Medical Genetics Research Institute	Assessment of attitudes and beliefs toward genetic tests and genetic research through questionnaire	General population (*N* = 2000)	<24 years: 27.4% 25–39 years: 43.2% 40–64 years: 27.9% ≥65 years: 1.5%
Molster et al. (2009)	Australia	Office of Population Health Genomics, Health Policy and Clinical Reform Division, Department of Health	Assessment of knowledge regarding human genetics and health by telephone survey	General population (*N* = 1009)	≥18 years
Morin (2009)	Canada	University	Assessment of knowledge and attitudes regarding nutrigenomics and nutrigenetic tests, through 12 focus groups lasting 2 h each preceded by a short questionnaire	General population (*N* = more than 90)	–
Stewart-Knox et al. (2009)	France, Italy, UK, Portugal, Poland, Germany	University	Assessment of attitudes toward genetic and nutrigenomics tests and personalized nutrition through a survey	General population (*N* = 5967)	≥14 years
Gleason et al. (2010)	USA	Course funded by Grant by U.S. Department of Education and GCSU Science Education Center	Improvement of genetic knowledge and awareness about ethical, legal, and social implications (ELSI) of Human Genome Project through an interdisciplinary course	Teachers (biology teachers *N* = 8; English teachers *N* = 8)	–
Hahn et al. (2010)	USA	University	Assessment of awareness and perceptions regarding genomics medicine and preferences related to educational strategies and contents through 13 focus groups lasting 1 h each	General population (*N* = 121)	–
Lemke et al. (2010)	USA	Funded by National Human Genome Research Institute	Assessment of attitudes and perceptions regarding collection and sharing of genetic research data, through 6 focus groups lasting 1–2 h each	General population (*N* = 28) and NUgene (biobank) participants (*N* = 21)	≥18 years
Sturgis et al. (2010)	UK	–	Assessment of the effect of information provided by a short film (extended version of 9 min and short version of 5 min and 40 s) on opinions regarding genomics through a survey	General population (1st phase *N* = 3270; 2nd phase *N* = 867; 3^rd^ phase *N* = 458)	–
Mai et al. (2011)	Greece	Partially funded by Golden Helix Institute of Biomedical Research and research budget of the University of Patras	Assessment of awareness and perceptions regarding issues related to genetics, genetic tests, and their impact on society through a survey	General population (*N* = 1717)	≥18 years
Smerecnik et al. (2011)	Netherlands	University	Assessment of knowledge about genetic risk factors of multifactorial diseases through online questionnaire	General population (*N* = 1624)	≥20 years
Dijkstra et al. (2012)	Netherlands	Financially supported by the Dutch Research Organization, in particular by the Societal and Ethical Aspects of Genomics program	Assessment of participation and attitudes toward genomics research and related problems through online survey	General population (*N* = 986), members of the public with experience in genomics research as patients (*N* = 41), patients with celiac disease (*N* = 68), experts (*N* = 45).	18–65 years
East et al. (2012)	USA	HudsonAlpha faculty	Improvement of genomics literacy through a short course (three editions), with assessment of learning through pre- and post-course tests	General population (1st course *N* = 110; 2nd course *N* = 86; 3^rd^ course *N* = 196; Total *N* = 392)	<20 years: 1.8% 21–30 years: 2.5% 31–40 years: 4.6% 41–50 years: 12.9% 51–60 years: 18.6% >60 years: 59.6%
Kaphingst et al. (2012)	USA	Supported by Intramural Research Program del National Human Genome Research Institute, National Institutes of Health	Assessment of knowledge about genome sequencing and of the influence of informed consent process on them (discussion with a geneticist lasting 60–90 min) through pre- and post-discussion questionnaires	ClinSeq™ participants (sequencing study) (*N* = 311)	–
Kolor et al. (2012)	USA	Lifestyle surveillance system	Assessment of awareness and use of DTC genetic tests through telephone survey	General population (Connecticut *N* = 6019; Michigan *N* = 5883; Oregon *N* = 1931; Utah *N* = 2606; national *N* = 5399)	≥18 years
Nielsen and El-Sohemy (2012)	Canada	University	Assessment of awareness, perceptions, and understanding of nutrigenomics and genetic tests through a questionnaire, and of the influence of personalized dietary advice based on the genotype on participants' opinions through a randomized trial	Toronto Nutrigenomics and Health Study (*N* = 149)	20–35 years
Bombard et al. (2013)	Canada	University-government research collaboration	Assessment of perspectives about ethical and social issues raised by personalized medicine and genetic tests through meetings preceded by an expert explanation	Citizens panel (*N* = 14)	18–71 years
Haga et al. (2013)	USA	University	Assessment of knowledge, attitudes, and expectations on health, genetics, and genetic tests, and of understanding and perceptions of genomic risk for Type 2 Diabetes Mellitus through 3 questionnaires	General population (*N* = 300)	≥18 years
Henneman et al. (2013)	Netherlands	Financially supported by Netherlands Genomics Initiative	Assessment of experiences, beliefs, and expectations regarding genetic tests over the years through surveys administered in 2002 and 2010	Consumers panel (2002 survey *N* = 817; 2010 survey *N* = 978)	25–90 years in 2002; 21–91 years in 2010
Nicholls et al. (2013)	Canada	University	Assessment of the usefulness perception of genomics applied to cancer and neonatal screening, through 8 workshops/seminars	General population (*N* = 170)	27–88 years
Almeling (2014)	USA	University	Assessment of opinions regarding policy issues in genetics and genomics through online survey, in which each participant was asked to respond imagining that he had an increased genetic risk for a specific disease	General population (*N* = 2100)	–
Borzekowski et al. (2014)	USA	University	Assessment of public reaction to the history of Angelina Jolie's preventive mastectomy and of the knowledge about breast cancer risk in carriers of mutations of the BRCA gene through online survey	General population (*N* = 2572)	≥18 years
Vermeulen et al. (2014)	Netherlands	University	Assessment of attitudes and interests toward genetic tests, in particular for prevention of chronic diseases, through a questionnaire	General population (*N* = 978)	≥18 years Mean: 59.1 years Range: 18–91 years
Waters et al. (2014)	USA	University	Assessment, through questionnaire, of: -attitudes in searching for information about chronic diseases and cancer -level of knowledge about their multifactorial etiopathogenesis and the role of genetic factors -relations between attitudes and knowledge	General population (*N* = 3630)	≥18 years
Abrams et al. (2015)	USA	Funded by Intramural Research Program of National Human Genome Research Institute	Assessment of literacy on genetics, genomics, and genetic tests through electronic questionnaires	Consumers panel representative of the adult population (*N* = 1016)	≥18 years
Dodson et al. (2015)	USA	University	Assessment of interest in whole-genome sequencing (WGS) through online survey, with specific questions for parents regarding their children	General population (parents and not) (*N* = 2144)	18–94 years
Etchegary et al. (2015)	Canada	University	Assessment of attitudes and expectations regarding genomics research through meetings (5 meetings lasting 1.5 h each) in which slides were shown to stimulate discussion	General population (*N* = 33)	≥18 years
Graves et al. (2015)	USA	Funded by Mayo Clinic Center for Individualized Medicine	Assessment, through online survey, of perceptions and attitudes regarding disease actionability and severity, and of experience and interest in knowing results of genetic tests	General population (*N* = 900)	18–70 years
Kaphingst et al. (2015)	USA	University	Assessment, through questionnaire, of effects of the type of risk assessment carried out (family history assessment or genetic test results), the type of disease (cardiovascular disease or diabetes), and ethnicity on the attitudes regarding the genetic risk for complex diseases and genetic testing	Medically underserved population (*N* = 1,057 females)	–
Mavroidopoulou et al. (2015)	Greece	University	Assessment of awareness, interest, motivation, and understanding of DTC genetic tests through questionnaire	University students, postgraduates and doctoral students (*N* = 725)	–
Kaphingst et al. (2016)	USA	University	Assessment, through survey, of the association between health literacy and knowledge, perceived importance, and attitudes on the communication of genetics, family history and genome sequencing	Medically underserved population (*N* = 624)	≥18 years
Mählmann et al. (2016)	Switzerland	University	Assessment, through a questionnaire, of attitudes toward personal genomics and genetic tests, after the view of a movie on the topic	Older adults attending university for the elderly (*N* = 151)	60–89 years
McCormack et al. (2016)	Europe	Supported by Medical Research Council UK and European Union Seventh Framework Programme	Assessment of attitudes regarding participation in genomics research, large-scale international databases, and sharing of biological samples, through 5 focus groups lasting 1–1.5 h each	Patients with rare diseases (*N* = 52)	–
Miyamoto et al. (2016)	Japan	University	Assessment of experiences and attitudes toward genomics and an ongoing genomic cohort study, through a postal questionnaire	General population (*N* = 1477)	30–69 years
Schmidlen et al. (2016)	USA	University	Assessment of knowledge on the association between genetic risk and complex diseases and between drug response and genetic susceptibility, through online questionnaire	General population (*N* = 2839), patients with prostate or breast cancer (*N* = 82), patients with hypertension or congestive heart failure (*N* = 201), medical and administrative staff (*N* = 940).	>18 years
Simonstein and Mashiach-Eizenberg (2016)	Israel	University	Assessment, through questionnaire, of attitudes toward genetic technologies, and their correlation with the understanding of genetics, reproduction and reproductive risk	Israeli Arabs and Jews (*N* = 203)	≥19 years
Waters et al. (2016)	USA	University	Assessment, through questionnaire, of attitudes in searching for information about cancer, of knowledge on its multifactorial etiopathogenesis and on the role of genetic factors, and of relation between attitudes and knowledge	General population (*N* = 2529)	≥18 years
Ahmed et al. (2017)	USA	–	Improvement of scientific and genetic literacy through Science Cafés held by experts	General population (Total *N* = 457; Health Science Cafés *N* = 248; Genomics Science Cafés *N* = 209)	19–39 years: 20.2% 40–59 years: 32% ≥60 years: 47.9%
Krakow et al. (2017)	USA	Government	Assessment, through survey, of knowledge and awareness of genetics, and of use of genetic tests	General population (*N* = 3285)	≥19 years
Waters et al. (2017)	USA	University	Identification of gaps in understanding and acceptance of research and genomics information through 13 focus groups, lasting 1–1.5 h each, during which a video with genetic information was shown and then participants were asked to discuss it	African American and white smokers (*N* = 84)	≥18 years
Fournier and Poulain (2018)	France	University	Assessment of knowledge and reactions toward nutritional genomics and epigenomics, food practices, food-health relation through 3 focus groups, lasting 2.5 h each	General population (*N* = 22)	Mean: 43 years 20–29 years: 9.1%; 30–39 years: 31.8%; 40–49 years: 31.8%; 50–59 years: 18.2%; ≥60 years: 9.1%
Metcalfe et al. (2018)	Australia	University	Assessment, through focus groups, of awareness, knowledge, attitudes and opinions regarding personal genetic tests	General population (*N* = 56)	≥18 years
Horrow et al. (2019)	USA	Supported by a grant of the National Human Genome Research Institute and the Mayo Clinic Center for Individualized Medicine	Development of a scale to evaluate attitudes about the future of genomics medicine (questionnaire)	Participants in a genomic sequencing study (*N* = 2895)	26–71 years
Jones et al. (2019)	UK	Funded by the UK Medical Research Council	Assessment, through a series of 8 public workshops and a questionnaire, of public views on access models for reusing genomics data collected for research in conjunction with health data	College students, university staff and students, business professionals, general public consumer panel, science festival attendees, health professionals, and University of Third Age members (*N* = 116)	16–25 years: 18.9%; 26–35 years: 31.8%; 36–45 years: 15.5%; 46–55 years: 9.4%; 56–65 years: 6.8%; >65 years: 17.2%
Pereira et al. (2019)	Korea, Canada, USA	Funded by National Institutes of Health (NIH)/National Heart, Lung, and Blood Institute (NHLBI) grant	Assessment of perceptions toward pharmacogenetic testing of patients undergoing percutaneous coronary intervention through a questionnaire (pre and post coronary intervention)	Patients undergoing percutaneous coronary intervention (Baseline survey: *N* = 1,327 Follow-up survey: *N* = 860)	Mean: 62.8 years ≤ 55 years: 27%; 56–65 years: 32%; 66–75 years: 27%; >75 years: 14%
Rebitschek et al. (2019)	Germany	Funded by the European Commission and supported by The Eve Appeal	Assessment, through 4 focus groups, of what women want to know about epigenetic cancer risk assessment, how they evaluate its usefulness, and how they would like to be informed about their risk	General population, women only (*N* = 25)	30–65 years
Middleton et al. (2020)	USA, UK, Canada, Australia	Supported by Well-come grant to the Society and Ethics Research Group, Connecting Science, Well-come Genome Campus, Cambridge, UK. Also supported by Global Alliance for Genomics and Health	Assessment, though a survey, of public perceptions of genomics data sharing	General population (*N* = 8,967)	≤ 30 years: 23.3%; 31–40 years: 22.8%; 41–50 years: 17.5%; 51–60 years: 17.7%; >60 years: 18.6%

The articles included in this systematic review were published between 2006 and 2020. Among the 54 included studies, 44% were conducted in USA (*n* = 24) (Fomous et al., 2006; Goddard et al., 2007, 2009; Gleason et al., 2010; Hahn et al., 2010; Lemke et al., 2010; East et al., 2012; Kaphingst et al., 2012, 2015, 2016; Kolor et al., 2012; Haga et al., 2013; Almeling, 2014; Borzekowski et al., 2014; Waters et al., 2014, 2016, 2017; Abrams et al., 2015; Dodson et al., 2015; Graves et al., 2015; Schmidlen et al., 2016; Ahmed et al., 2017; Krakow et al., 2017; Horrow et al., 2019), 22% (*n* = 12) in the European Union (EU27) (Henneman et al., 2006, 2013; Calsbeek et al., 2007; Morren et al., 2007; Mai et al., 2011; Smerecnik et al., 2011; Dijkstra et al., 2012; Vermeulen et al., 2014; Mavroidopoulou et al., 2015; McCormack et al., 2016; Fournier and Poulain, 2018; Rebitschek et al., 2019), 9% (*n* = 5) in Canada (Morin, 2009; Nielsen and El-Sohemy, 2012; Bombard et al., 2013; Nicholls et al., 2013; Etchegary et al., 2015), 6% (*n* = 3) in UK (Skirton et al., 2006; Sturgis et al., 2010; Jones et al., 2019), 6% (*n* = 3) in various countries (Stewart-Knox et al., 2009; Pereira et al., 2019; Middleton et al., 2020), 4% (*n* = 2) in Australia (Molster et al., 2009; Metcalfe et al., 2018), 4% (*n* = 2) in Japan (Ishiyama et al., 2008; Miyamoto et al., 2016), 2% (*n* = 1) in Israel (Simonstein and Mashiach-Eizenberg, 2016), 2% (*n* = 1) in Russia (Makeeva et al., 2009) and, eventually, 2% (*n* = 1) in Switzerland (Mählmann et al., 2016).

Regarding the target population, most of studies (76%) addressed samples representative of the general population (Fomous et al., 2006; Henneman et al., 2006, 2013; Goddard et al., 2007, 2009; Ishiyama et al., 2008; Makeeva et al., 2009; Molster et al., 2009; Morin, 2009; Stewart-Knox et al., 2009; Hahn et al., 2010; Lemke et al., 2010; Sturgis et al., 2010; Mai et al., 2011; Smerecnik et al., 2011; Dijkstra et al., 2012; East et al., 2012; Kolor et al., 2012; Nielsen and El-Sohemy, 2012; Bombard et al., 2013; Haga et al., 2013; Nicholls et al., 2013; Almeling, 2014; Borzekowski et al., 2014; Vermeulen et al., 2014; Waters et al., 2014, 2016; Abrams et al., 2015; Dodson et al., 2015; Etchegary et al., 2015; Graves et al., 2015; Miyamoto et al., 2016; Schmidlen et al., 2016; Simonstein and Mashiach-Eizenberg, 2016; Ahmed et al., 2017; Krakow et al., 2017; Fournier and Poulain, 2018; Metcalfe et al., 2018; Jones et al., 2019; Rebitschek et al., 2019; Middleton et al., 2020), while the remaining part involved samples selected based on demographic characteristics (detailed description in Table 1).

In most cases (59%) the initiatives described were promoted by universities (Fomous et al., 2006; Henneman et al., 2006; Skirton et al., 2006; Morren et al., 2007; Morin, 2009; Stewart-Knox et al., 2009; Hahn et al., 2010; Mai et al., 2011; Smerecnik et al., 2011; Nielsen and El-Sohemy, 2012; Bombard et al., 2013; Haga et al., 2013; Nicholls et al., 2013; Almeling, 2014; Borzekowski et al., 2014; Vermeulen et al., 2014; Waters et al., 2014, 2016, 2017; Dodson et al., 2015; Etchegary et al., 2015; Graves et al., 2015; Kaphingst et al., 2015, 2016; Mavroidopoulou et al., 2015; Mählmann et al., 2016; Miyamoto et al., 2016; Schmidlen et al., 2016; Simonstein and Mashiach-Eizenberg, 2016; Fournier and Poulain, 2018; Metcalfe et al., 2018).

Overall, 72% of included studies (*n* = 39) investigated citizens' knowledge (Table 2), 56% (*n* = 30) attitudes (Table 3), and 20% (*n* = 11) educational needs (Table 2) in the field of omics sciences, while 52% (*n* = 28) investigated attitudes and perceptions about genetic and/or omics tests (Table 4). To this end, tools such as questionnaires or surveys were used in 74% (*n* = 40) of studies (Henneman et al., 2006, 2013; Calsbeek et al., 2007; Goddard et al., 2007, 2009; Morren et al., 2007; Ishiyama et al., 2008; Makeeva et al., 2009; Molster et al., 2009; Stewart-Knox et al., 2009; Sturgis et al., 2010; Mai et al., 2011; Smerecnik et al., 2011; Dijkstra et al., 2012; East et al., 2012; Kaphingst et al., 2012, 2015, 2016; Kolor et al., 2012; Nielsen and El-Sohemy, 2012; Haga et al., 2013; Almeling, 2014; Borzekowski et al., 2014; Vermeulen et al., 2014; Waters et al., 2014, 2016; Abrams et al., 2015; Dodson et al., 2015; Etchegary et al., 2015; Graves et al., 2015; Mavroidopoulou et al., 2015; Mählmann et al., 2016; Miyamoto et al., 2016; Schmidlen et al., 2016; Simonstein and Mashiach-Eizenberg, 2016; Krakow et al., 2017; Horrow et al., 2019; Jones et al., 2019; Pereira et al., 2019; Middleton et al., 2020). Instead, 26% (n =14) reported residential methods, such as meetings, workshop, or focus groups (Skirton et al., 2006; Morin, 2009; Hahn et al., 2010; Lemke et al., 2010; Bombard et al., 2013; Nicholls et al., 2013; Etchegary et al., 2015; McCormack et al., 2016; Ahmed et al., 2017; Waters et al., 2017; Fournier and Poulain, 2018; Metcalfe et al., 2018; Jones et al., 2019; Rebitschek et al., 2019). Furthermore, in 4% of the studies (*n* = 2) a course was developed (Gleason et al., 2010; East et al., 2012), while in 4% of cases (*n* = 2) a movie or a short video was realized (Sturgis et al., 2010; Mählmann et al., 2016). Lastly, one study (2%) involved the online consultation of a web portal on genomics and its implications (Fomous et al., 2006), and in another one (2%), focused on nutrigenomics, a clinical trial was carried out (Nielsen and El-Sohemy, 2012).

**Table 2:** Citizens' knowledge and educational needs in the field of omics sciences (where investigated).

**References**	**Awareness and knowledge about genomics/omics sciences**	**Factors affecting knowledge**	**Request or need for more education/information regarding genomics/omics sciences by the population**
Skirton et al. (2006)	Very low. Participants were largely unsure about the underlying scientific basis of genetics.	–	Participants were keen to learn more.
Calsbeek et al. (2007)	Low, heterogeneous. Better knowledge on association between genes and diseases. Perceived knowledge about genetic testing did not increase from 2002 to 2004.	–	–
Goddard et al. (2007)	Low. 14% of participants aware of DTC nutrigenomics tests; 15% aware of pharmacogenetic tests; 30% aware of genetic tests to assess disease risk; 38% aware of genetic tests to diagnose diseases. Main sources of information on DTC nutrigenetic tests: television (46%), magazine (35%), newspapers (29%).	–	–
Morren et al. (2007)	Low. Most of the respondents reported having poor knowledge of genetics (about 10% reported having knowledge about it, while half to three quarters indicated that they had no knowledge on the topic).	–	–
Ishiyama et al. (2008)	Low, heterogeneous. Extremely variable level of knowledge and understanding (the percentage of correct answers varies between 12.7% and 71.9% depending on the question). Most of participants approve the promotion of genomics studies.	–	–
Goddard et al. (2009)	Low. Estimates of awareness about direct-to-consumer nutrigenomics test ranged from 7.6% in Michigan to 24.4% in Oregon.	–	–
Molster et al. (2009)	Low. 46% said they knew very little about human genes and health.	–	–
Morin (2009)	Low. Limited knowledge about current nutrigenomics practices.	–	More public education on nutrigenomics needed.
Gleason et al. (2010)	Low, heterogeneous. Before the course, Biology teachers had better knowledge, while English ones very poor knowledge. After the course, participants showed significant improvements in knowledge about genetics and the Human Genome Project.	–	–
Hahn et al. (2010)	Low. Most participants never even heard the term “genomics medicine.” Most were unfamiliar with the term “personalized medicine.”	–	Willingness to know more about specific diseases, use in research, cloning and genetic engineering.
Lemke et al. (2010)	Low, heterogeneous. Variable and scarce knowledge on genomics research: in most cases participants associated genetic research only with diseases, few knew of basic research.	–	Willingness to receive more information on genetic research, as it was considered important for reducing fears and increasing confidence. It is important to increase awareness in isolated groups (low income, minorities), young people, schools, neighborhoods and disease support groups. Suggested strategy was to target populations at greater risk for a given condition than the general population. Other proposed strategies were information via evening TV news, internet, focus groups.
Mai et al. (2011)	Moderate. Most of participants were aware of simple concepts of general genetics and genetic testing.	Poorer knowledge with increasing age and in small towns compared to cities.	–
Smerecnik et al. (2011)	Low. 17.9% said they heard of genetic risk factors for multifactorial diseases.	–	–
Dijkstra et al. (2012)	Low. Poor knowledge about genomics research (24.2% never read information on genomics research, 54.1% sometimes).	–	Most of participants never searched for information about genomics research (71.4% never searched for information on the internet or library).
East et al. (2012)	Low/very low before the course. Course led to a significant improvement of perceived knowledge	–	Participants reported a high likelihood for continued self-learning after the course.
Kaphingst et al. (2012)	Heterogeneous. Level of knowledge on limits and benefits of sequencing was very variable before starting the study. Knowledge increased significantly from pre to post informed consent.	Level of knowledge related to the level of education.	–
Kolor et al. (2012)	Heterogeneous. DTC-GT awareness ranged from 15.8% in Michigan to 29.1% in Oregon. The most commonly cited source from which respondents read or heard of DTC-GTs was, in descending order, TV or radio, newspaper/magazine and Internet.	–	–
Nielsen and El-Sohemy (2012)	Low. Fifty-two percentage said they had not heard “anything” about DTC genetic testing.	–	–
Bombard et al. (2013)	–	–	Participants called for increased public awareness about personalized medicine to increase confidence and use of new technologies, in addition to counseling services.
Haga et al. (2013)	Moderate. Participants scored significantly higher on questions related to heredity and causes of diseases (average score of 94.6%) compared to questions on genes, chromosomes and cells (average score of 78.6%). Most of participants (79%) said they had some knowledge of the medical applications of genetics.	–	–
Henneman et al. (2013)	Low. Awareness about genetic testing and genetic diseases did not change between 2002 and 2010.	–	–
Almeling (2014)	Low. About a fifth of respondents heard of companies selling genetic tests directly to consumers.	–	–
Borzekowski et al. (2014)	Low. Less than 10% knew the average risk of breast cancer in the general population and the fraction of breast cancers due to BRCA mutations.	Knowledge of Angelina Jolie's story was not associated with better understanding.	–
Waters et al. (2014)	Moderate-high. Most of participants (64.2–78.6%) reported awareness of the multifactorial etiopathogenesis of diseases.	–	–
Abrams et al. (2015)	Moderate. Participants were “somewhat familiar” with genomics terms presented. Average score in the assessment of skills resulted 4 out of 6 correct answers, while on average participants correctly identified 8 out of 16 facts.	Level of knowledge related to the level of education.	–
Dodson et al. (2015)	Moderate. About 58.6% of respondents expressed some interest in whole genome sequencing, especially in those who expressed interest in having a child in the next 5 years.	–	–
Etchegary et al. (2015)	Low/very low. Many participants noted they lacked knowledge about genetics and associated research.	–	Participants reported the necessity for accurate information to make informed decisions both about genetic testing and participation in genetics research.
Kaphingst et al. (2015)	–	–	Strong interest in receiving a genomics evaluation for reference diseases (diabetes/heart disease), in discussing genomics information with family members and a doctor, and in modifying lifestyle in relation to genomics information.
Mavroidopoulou et al. (2015)	Low. 43.7% of the participants had the perception of being lacking in basic knowledge of genetics, unlike 34.5% who believed they knew enough.	–	–
Kaphingst et al. (2016)	Moderate. On average, participants answered 3 out of 5 questions regarding genomics correctly.	Poorer knowledge in the elderly than in the youth.	–
Mählmann et al. (2016)	Low. One third of respondents reported having heard of personal genetic tests.	–	–
Miyamoto et al. (2016)	Moderate. Adopted scale and frequency of participants in each group: “High” level of knowledge: understanding of the terms genome and gene or understanding of the term “gene” and having heard of “genome” = 30.2%; “Medium” level: having heard of both terms or understanding the term “gene” and having never heard of “genome” = 35.7%; “Low” knowledge consisted of having heard of “gene” and not of “genome” or of never having heard of both terms = 34.1%.	–	–
Schmidlen et al. (2016)	Moderate-high. Average genetic knowledge score among participants: 76%.	Higher level of knowledge in those who had previous experiences in genetic education (genetics courses, website consultations and reading of books and/or articles).	–
Simonstein and Mashiach-Eizenberg (2016)	Low. Most participants did not understand basic level questions in the general understanding of genetics section. 47.6% correct answers in the general understanding section of genetics. 74.9% correct answers in the genetic risk section.	–	–
Waters et al. (2016)	Moderate. Participants had relatively high previous experience with cancer, through family history (66.5%) or awareness about DTC genetic tests (51.1%).	–	–
Ahmed et al. (2017)	Low. However, Science Café model led to a change in participants' perceived literacy level.	Individuals with middle or high socioeconomic status perceived a greater change, while a level of education beyond high school was associated with greater difficulty in increasing literacy.	–
Krakow et al. (2017)	Moderate, referred to genetic tests. 57% of participants were aware of genetic tests.	–	–
Waters et al. (2017)	Low. Beliefs were not always consistent with biomedical explanations about the relationship between genes and addiction.	–	–
Fournier and Poulain (2018)	–	–	Many doubts about nutrigenetic tests and, therefore, a need for information on regulation, scientific evidence, medical support, and costs/economy was expressed.
Metcalfe et al. (2018)	Low. Knowledge about personal genomics tests was poor, but most participants could guess what the term “personal genomics” could imply. A minority had heard the term “direct-to-consumer test.”	–	–
Horrow et al. (2019)	High. Mean score in the genomic knowledge (knowledge of sequencing limitations and potential benefits) was 8.1 out of 11.00.	–	–
Jones et al. (2019)	Low. 6.8% of participants reported no knowledge of genetics, 34.4% little, 36.2% middling, 14.6% good, and 6.8% very good.	–	–
Rebitschek et al. (2019)	–	–	Study participants wanted to understand how the epigenetic approach is different from established genomics tests, how epigenetic changes relate to cancer, and whether the test enables monitoring of one's cancer risk. Furthermore, they wanted to know more about basic cancer risks and information after epigenetic testing about non-invasive preventive options regarding both health care and preventive behavior.

**Table 3:** Citizens' attitudes on omics sciences (where investigated).

**References**	**Perception of benefits in the use of genetics/omics sciences in medicine**	**Concerns about the use of genetics/omics sciences in medicine**
Henneman et al. (2006)	Two hundred and sixty-four supporters vs. 248 against the availability and use of genetic tests. Forty-three percentage believed that knowing genetic background of diseases would help people live longer.	Among respondents, 40% thought that knowing own genetic makeup would be deterministic for people because it would decrease their self-esteem and/or deprive people of the freedom to live as desired. According to 44% of the interviewees, tests could lead to social exclusion of subjects with positive tests and handicaps.
Skirton et al. (2006)	Enhanced medical knowledge could enable preventive measures to be taken.	Ethical issues, psychological consequences of genetic test results.
Calsbeek et al. (2007)	–	Some participants considered genetic tests “frightening”; some were concerned about insurance consequences of the tests.
Morren et al. (2007)	Most of the interviewees (70–80%) approved genetic tests and considered genetic research important for future treatment of diseases.	Especially consequences in case of positivity to genetic tests for work and medical insurance.
Ishiyama et al. (2008)	Genomic literacy related with positive attitude toward genomic studies.	–
Makeeva et al. (2009)	Among respondents, 81.3% believed that genetics would help people live longer and 81.4% that would help keep their lifestyle under control.	Among the participants, 48.0% were worried about discrimination based on genetic data.
Morin (2009)	Consumers believed that potential benefits of nutrigenomics outweigh risks. For participants, nutrigenomics could also lead to early diagnosis or disease prevention and, in general, could encourage healthy eating habits.	Toward online services and DTC genetic tests.Greater regulatory control is needed to protect consumers (in particular for sale of genetic tests).
Stewart-Knox et al. (2009)	Perceived benefits of nutrigenomics to follow a personalized diet.	Concerns about how information would be used.
Hahn et al. (2010)	For disease prevention and treatment.	Affordability, unanticipated physical harm, mistrust of the government and researchers, downstream effects like overpopulation, playing God/disturbing the natural order, lack of regulations, loss of privacy, genetic discrimination, and moral dilemmas posed by genetic engineering, cloning, choosing traits, and abortions resulting from genetic information.
Lemke et al. (2010)	Among perceived benefits of genomics research there were the possibility to prevent and treat diseases and potential cost savings to society.	Concerns about sharing research results with the public, possibility of discrimination by insurance companies, the government, the health care system, and employers.
Nielsen and El-Sohemy (2012)	Nutrigenomics information was considered useful for motivation to change lifestyle (prevention).	–
Bombard et al. (2013)	For disease prediction and treatment.	Costs, accessibility, need, and feasibility of introduction into the health system.
Haga et al. (2013)	More than half of participants agreed with the possibility that a DNA test will change a person's future (56.3%).	More than half of participants agreed with the possibility that a DNA test will affect a person's ability to obtain health insurance (51.3%), 16% were worried about the consequences of tests on the possibility to find a job.
Henneman et al. (2013)	–	Some believed that insurance companies will require genetic testing in the future to determine the premium (36%, <2002).
Nicholls et al. (2013)	The application of genomics to screenings could allow early intervention, prevention, and stricter monitoring.	Costs, educational needs regarding the probabilistic nature of the risk, access to personal genomics information.
Almeling (2014)	Perception of importance/utility (indirectly: 57% agree that the federal government should spend more on researching the genetic causes of diseases).	Important to avoid discrimination (82% believe GINA–Genetic Information Non-discrimination Act 2008–is important).
Vermeulen et al. (2014)	Especially interests for preventive genomics.	–
Etchegary et al. (2015)	Omics sciences perceived as beneficial, but not a priority for the health system.	Dangerous use of information, privacy issues.
Kaphingst et al. (2015)	Black women perceived fewer health benefits than white women; Hispanics had greater interest in receiving a genomics assessment than non-Hispanic whites.	–
Mavroidopoulou et al. (2015)	Most participants expressed interest in undergoing DTC genetic testing for serious diseases, such as cancer (54.9%), or a metabolism or genealogy test (50.2%), but would prefer to consult their doctor first.	Concerns about personal data.
Kaphingst et al. (2016)	Fifty four percentage of participants considered genetic information very important.	–
Mählmann et al. (2016)	Participants were interested in personal genomics mostly to find out about their own disease risk or to contribute to scientific research.	Concerns and doubts about psychological implications deriving from testing results, validity of tests, privacy issues.
McCormack et al. (2016)	Participants understood the importance of sharing data and samples and creating databases for rare diseases.	Concerns about risks to privacy and autonomy in sharing data on an international database. Need to limit access to personal data to health professionals involved in research. All participants were against access to data by private companies.
Miyamoto et al. (2016)	Over 80% of participants agreed that the use of genetic information for medical purposes is “useful for disease diagnosis,” “useful for disease treatment” and “useful for disease prevention”	Less than half of respondents showed “concerns” about the use of genomics in medicine (worries regarded the use of genomics information by companies or government agencies, possible discrimination at work and by insurance companies, problems related to cloning of humans, unexpected negative effects).
Simonstein and Mashiach-Eizenberg (2016)	Most (81.3%) believed that medicine could improve with the use of genetic engineering.	Almost half of the participants agreed (22.7% partially agreed, 18.7% agreed) with the sentence “genetic engineering could destroy the human race.”
Fournier and Poulain (2018)	Nutritional epigenomics was perceived positively compared to nutritional genomics.	Possible conflicts of interest between scientific research and the agri-food industry.
Metcalfe et al. (2018)	Only for health purposes.	Concerns about privacy issues, potential discrimination. Cost skepticism (speculation by companies) was expressed too.
Horrow et al. (2019)	Genomic optimism was positively associated with higher health literacy.	Genomics pessimism was associated with lower health literacy.
Jones et al. (2019)	The majority (67.2%) of questionnaire respondents placed a high or very high value on using genetic data for research.	Almost half of the respondents reported moderate concern about the use of genetic data for research (48%). Most commonly expressed concern was misuse of data tied with concerns about information governance. Participants expressed the importance of informed consent for access to genetic data for research and a preference for a safe use of the data.
Middleton et al. (2020)	–	The main potential harm identified was the possibility to use DNA of other individuals to blame them for a crime (45.2%), insurance discriminations (37.2%), use of genetic information by companies for targeted marketing strategies (35%).

**Table 4:** Citizens' perceptions and attitudes about genetic and/or omics tests (where investigated).

**References**	**Perceptions about genetic/omics tests**
Henneman et al. (2006)	Variable, almost half of respondents neither agreed nor disagreed with the statement that genetic testing carries more benefits than risks.
Skirton et al. (2006)	Positive, in particular for disease prevention and prevention for family members.
Calsbeek et al. (2007)	Positive, most participants approved genetic testing for the treatment of diseases (78% to 86%) or for early detection of diseases. However, some concerns were expressed, mostly related to the consequences of DNA-testing for taking out insurances.
Morren et al. (2007)	Variable. Just over two thirds said they would like to know if their disease is genetic. Thirty percentage said they did not want to know if they are at risk of a genetic disorder. If no appropriate treatment was available, over 40% of chronic patients would abstain from genetic test. Most respondents thought that the family should be informed of test results and would share the results with their children (70%) and siblings (65%).
Makeeva et al. (2009)	Positive, most participants stated that they would undergo tests and change their lifestyle based on the results.
Morin (2009)	Positive, nutrigenomics tests could have a positive impact on behavior/lifestyle. Worries about online purchase of genetic tests. Participants expressed a clear preference for in-person testing at a clinic or laboratory, for direct interaction with a healthcare professional.
Stewart-Knox et al. (2009)	Positive, 66% of participants said they would undergo a genetic test.
Hahn et al. (2010)	Variable/with doubts. Positive but with some skepticism.
Mai et al. (2011)	Positive, most participants stated that they would undergo a test even if not reimbursed. Most were against DTC genetic tests, most believed that a doctor should prescribe them and explain results.
Nielsen and El-Sohemy (2012)	Positive, nutrigenomics knowledge was considered useful to motivate people to change own lifestyle.
Bombard et al. (2013)	Variable/with doubts. Positive, but concerns for ethical issues for access to treatments based on genetics were expressed.
Haga et al. (2013)	Variable. 52% of participants stated they were interested in genetic testing and 45% said they were extremely interested. Most participants expressed positive attitudes toward the goals of genetic research and the use of genetic tests.
Henneman et al. (2013)	Variable, not everyone agreed that genetic tests make it possible to live longer or that they carry more benefits than harms. In 2010 more people (compared to 2002) believed that the use of genetic tests should be promoted, although 37% did not agree.
Nicholls et al. (2013)	Variable, basically positive attitudes.
Almeling (2014)	Most believed that healthcare professionals should be involved in explaining test results.
Vermeulen et al. (2014)	Variable. About half of the participants expressed interest in genetic testing for prevention of specific diseases (cancer, cardiovascular disease, diabetes, or dementia). According to participants, genetic tests should be performed in hospitals (66%) and addressed to curable (57%) or preventable (69%) diseases. Older participants said they wanted to know only about diseases that can be treated more often than younger ones (65% vs. 47%).
Etchegary et al. (2015)	Controversial, with concerns mostly related to data privacy and possible harmful uses of test results.
Graves et al. (2015)	Variable. The existence of a treatment (87%) and the severity of the disease (85%) were considered important in deciding which test results one wants to know.
Kaphingst et al. (2015)	Variable. Greater interest in genomics assessments among those with a limited health literacy.
Mavroidopoulou et al. (2015)	Positive. Genetic tests must be preceded by medical genetic counseling. Interest in undergoing a DTC genetic test was cost dependent.
Mählmann et al. (2016)	Positive, most participants were interested in undergoing genetic testing to know their own disease risk.
Miyamoto et al. (2016)	Variable. Positive toward genetic tests for drug susceptibility (48.5% of the interviewees showed positive attitudes, 29.7% did not know how to respond and 21.7% showed negative attitudes).
Simonstein and Mashiach-Eizenberg (2016)	Positive, most supported using genetic testing during pregnancy (86.7% believed that screening for genetic risk in potential parents is not wrong; 72.9% believed that all women planning a pregnancy should undergo a genetic test)
Fournier and Poulain (2018)	Mostly negative. Some interests toward nutrigenetic tests have been identified (for curiosity, scientific progress, early diagnosis), but much of the discussion has shown reluctance toward their adoption. Worries for dangerous use of the internet, criticism of science, deterministic aspect, fear of knowing, desire/right not to know, ethical issues, attachment to French food models were expressed.
Metcalfe et al. (2018)	Variable. Heterogeneous among participants. Positive attitude for tests for health conditions, especially for diseases for which there is treatment/cure.
Horrow et al. (2019)	Mostly positive. Most respondents attributed high value to their potential genetic test results. Some participants (35.2%) did express concern about the confidentiality of their genetic test results.
Pereira et al. (2019)	Mostly positive. Most patients (77%) were interested in finding out if they had pharmacogenetic variants or other genetic variants that were related to their health (73%).
Rebitschek et al. (2019)	Variable about epigenetic testing aimed to assess personal cancer risk. Participants' arguments in favor of epigenetic cancer risk assessment covered three major beliefs: guidance on one's individual medical strategy, the development of coping strategies (empowerment) by knowing one's risks, and a motivational push to healthier or more conscious living. Unnecessary worry about cancer risk and consequences, the uncertainty surrounding the test result, a perceived lack of test benefit were the main concerns that were brought up against testing.

Finally, all the studies included in our systematic review deal with topics related to genomics and genetics or genetic tests, while we found none related to omics in general or to other specific branch of omics sciences such as metabolomics or proteomics.

### Knowledge

Among studies investigating citizens' knowledge, 31% (*n* = 12) aimed to evaluate knowledge on basic concepts in genetics among the general population (Fomous et al., 2006; Skirton et al., 2006; Calsbeek et al., 2007; Ishiyama et al., 2008; Molster et al., 2009; Sturgis et al., 2010; Mai et al., 2011; East et al., 2012; Abrams et al., 2015; Miyamoto et al., 2016; Simonstein and Mashiach-Eizenberg, 2016; Jones et al., 2019) (Table 2). More than half of the identified studies (72%, *n* = 28), instead, investigated knowledge of the population on genetic and/or genomics tests (Henneman et al., 2006, 2013; Skirton et al., 2006; Goddard et al., 2007, 2009; Morren et al., 2007; Makeeva et al., 2009; Stewart-Knox et al., 2009; Mai et al., 2011; Kaphingst et al., 2012; Kolor et al., 2012; Haga et al., 2013; Nicholls et al., 2013; Almeling, 2014; Vermeulen et al., 2014; Abrams et al., 2015; Dodson et al., 2015; Graves et al., 2015; Mavroidopoulou et al., 2015; Mählmann et al., 2016; Krakow et al., 2017; Metcalfe et al., 2018; Horrow et al., 2019) (Table 2).

Thirty-eight percent of the studies (*n* = 15) evaluated citizens' knowledge regarding the role of genetic factors in disease risk (including reproductive risk/fetal disease risk) (Morren et al., 2007; Makeeva et al., 2009; Molster et al., 2009; Hahn et al., 2010; Smerecnik et al., 2011; Nicholls et al., 2013; Borzekowski et al., 2014; Vermeulen et al., 2014; Waters et al., 2014, 2016, 2017; Abrams et al., 2015; Graves et al., 2015; Schmidlen et al., 2016; Simonstein and Mashiach-Eizenberg, 2016) (Table 2).

Furthermore, 15% of the studies (*n* = 6) addressed the ethical, social and legal implications related to genetic information (Fomous et al., 2006; Makeeva et al., 2009; Gleason et al., 2010; Lemke et al., 2010; Bombard et al., 2013; Almeling, 2014; McCormack et al., 2016). Ten percent (*n* = 4) of included articles focused on nutrigenomics and/or nutrigenetic tests (Goddard et al., 2007, 2009; Morin, 2009; Nielsen and El-Sohemy, 2012). Lastly, 10% of the studies (*n* = 4) dealt with the topic of genomics research (Ishiyama et al., 2008; Lemke et al., 2010; Dijkstra et al., 2012; Etchegary et al., 2015).

Study participants had, in most cases (64%, *n* = 25), a limited knowledge level regarding the abovementioned topics (Skirton et al., 2006; Calsbeek et al., 2007; Goddard et al., 2007, 2009; Morren et al., 2007; Ishiyama et al., 2008; Molster et al., 2009; Morin, 2009; Gleason et al., 2010; Hahn et al., 2010; Lemke et al., 2010; Smerecnik et al., 2011; Dijkstra et al., 2012; East et al., 2012; Nielsen and El-Sohemy, 2012; Almeling, 2014; Borzekowski et al., 2014; Abrams et al., 2015; Etchegary et al., 2015; Mavroidopoulou et al., 2015; Mählmann et al., 2016; Simonstein and Mashiach-Eizenberg, 2016; Ahmed et al., 2017; Waters et al., 2017; Metcalfe et al., 2018; Jones et al., 2019) (Table 2). Instead, few studies (26%, *n* = 10) showed a medium or moderate knowledge level (Henneman et al., 2006; Molster et al., 2009; Haga et al., 2013; Waters et al., 2014, 2016; Abrams et al., 2015; Dodson et al., 2015; Kaphingst et al., 2016; Miyamoto et al., 2016; Krakow et al., 2017). Eventually, just a small number of included studies (10%, *n* = 4) proved a medium-high or high knowledge level (Waters et al., 2014, 2016; Schmidlen et al., 2016; Horrow et al., 2019).

Several studies included in our review reported a correlation between citizens' level of knowledge and some of their socio-demographic characteristics. Mai et al. reported that individuals living in villages had poorer awareness about the existence (61.5%) and the biological role (16.9%) of DNA compared to individuals living in cities (93.7 and 80.8% respectively) (Mai et al., 2011). A similar trend was observed for age, with respondents aged <35 years more likely to be aware of the existence (94.6%) and the biological role (85.5%) of DNA compared to individuals older than 60 (86.6 and 60% respectively) (Mai et al., 2011). Kaphingst et al. reported a significantly lower genetic knowledge among older participants compared to younger participants, but also that Black participants had lower genetic knowledge than White participants (Kaphingst et al., 2016). Lastly, 2 studies investigated the correlation between citizens' educational attainment and the effects on genetic literacy. In particular, the first one reported that knowledge about limitations of genome sequencing was higher among individuals with any post-graduate education (odds ratio, OR: 8.7; 95% confidence interval, CI: 2.45–31.10) and with a college degree (OR: 3.9; 95% CI: 1.05–14.61) compared to individuals with an education level lower than a college degree (Kaphingst et al., 2012). Instead, the last one reported a correlation (Pearson correlation coefficient, r: 0.357; *p* < 0.001) between higher education level (ranging from less than high school to bachelor or higher) and better genetic knowledge (Abrams et al., 2015).

#### Sources of Citizens' Information

Two studies (5%) investigated the main sources of citizens' information (Goddard et al., 2007; Kolor et al., 2012; Table 2). Goddard et al. in a study published in 2007 and focused on DTC nutrigenetic tests, pointed out that TV (for 46% of survey respondents), magazines (35%), and newspaper (29%) were the major sources of information among study participants. Similarly, Kolor et al. (2012) showed that most commonly interviewed people read or listened about DTC-GTs on TV or radio, newspapers or magazines, and internet, in this order.

### Attitudes

The topic of citizens' perceptions about benefits and risks related to the use of genomics or omics sciences in medicine was investigated by 56% (*n* = 30) of the studies included (Henneman et al., 2006, 2013; Skirton et al., 2006; Calsbeek et al., 2007; Morren et al., 2007; Ishiyama et al., 2008; Makeeva et al., 2009; Morin, 2009; Stewart-Knox et al., 2009; Hahn et al., 2010; Lemke et al., 2010; Nielsen and El-Sohemy, 2012; Bombard et al., 2013; Haga et al., 2013; Nicholls et al., 2013; Almeling, 2014; Vermeulen et al., 2014; Etchegary et al., 2015; Kaphingst et al., 2015, 2016; Mavroidopoulou et al., 2015; Mählmann et al., 2016; McCormack et al., 2016; Miyamoto et al., 2016; Simonstein and Mashiach-Eizenberg, 2016; Fournier and Poulain, 2018; Metcalfe et al., 2018; Horrow et al., 2019; Jones et al., 2019; Middleton et al., 2020; Table 3). Most of them (59%, *n* = 16) showed that participants perceived benefits and usefulness from the use of genomics and omics science in medicine (Skirton et al., 2006; Morren et al., 2007; Makeeva et al., 2009; Morin, 2009; Stewart-Knox et al., 2009; Hahn et al., 2010; Lemke et al., 2010; Nielsen and El-Sohemy, 2012; Bombard et al., 2013; Nicholls et al., 2013; Almeling, 2014; Mählmann et al., 2016; McCormack et al., 2016; Miyamoto et al., 2016; Simonstein and Mashiach-Eizenberg, 2016; Metcalfe et al., 2018). In detail, perceived benefits concentrated in the fields of prevention (lifestyle modification) and treatment (personalization of treatment) (Morren et al., 2007; Morin, 2009; Hahn et al., 2010; Nielsen and El-Sohemy, 2012; Bombard et al., 2013; Nicholls et al., 2013; Miyamoto et al., 2016), and in general were related to a potential improvement in life expectancy (Henneman et al., 2006; Makeeva et al., 2009).

The vast majority of study participants report concern on the privacy and use, sharing and access to data (Stewart-Knox et al., 2009; Lemke et al., 2010; Nicholls et al., 2013; Etchegary et al., 2015; Mavroidopoulou et al., 2015; Mählmann et al., 2016; Miyamoto et al., 2016; Metcalfe et al., 2018; Jones et al., 2019; Middleton et al., 2020), and job discriminations (Henneman et al., 2006; Makeeva et al., 2009; Lemke et al., 2010; Almeling, 2014; Metcalfe et al., 2018) or insurance issues (Calsbeek et al., 2007; Morren et al., 2007; Lemke et al., 2010; Haga et al., 2013; Henneman et al., 2013; Miyamoto et al., 2016; Middleton et al., 2020). Three studies (Morren et al., 2007; Haga et al., 2013; Miyamoto et al., 2016) reported public's concern regarding potential consequences of genetic testing results, or in general personal genomics information, on working and professional life. Further issues and concern included costs (Hahn et al., 2010; Bombard et al., 2013; Nicholls et al., 2013; Metcalfe et al., 2018), access to tests and feasibility of the introduction of genetic tests in the health service (Bombard et al., 2013), and psychological distress, fears, and anxiety potentially caused by genetic test results (Skirton et al., 2006; Mählmann et al., 2016).

Regarding perceptions on genetic or omics tests (Table 4), investigated by 52% (*n* = 28) of the studies (Henneman et al., 2006, 2013; Skirton et al., 2006; Calsbeek et al., 2007; Morren et al., 2007; Makeeva et al., 2009; Morin, 2009; Stewart-Knox et al., 2009; Hahn et al., 2010; Mai et al., 2011; Nielsen and El-Sohemy, 2012; Bombard et al., 2013; Haga et al., 2013; Nicholls et al., 2013; Almeling, 2014; Vermeulen et al., 2014; Etchegary et al., 2015; Graves et al., 2015; Kaphingst et al., 2015; Mavroidopoulou et al., 2015; Mählmann et al., 2016; Miyamoto et al., 2016; Simonstein and Mashiach-Eizenberg, 2016; Fournier and Poulain, 2018; Metcalfe et al., 2018; Horrow et al., 2019; Pereira et al., 2019; Rebitschek et al., 2019), 39% of them highlighted overall positive attitudes among involved individuals (Skirton et al., 2006; Calsbeek et al., 2007; Makeeva et al., 2009; Morin, 2009; Stewart-Knox et al., 2009; Mai et al., 2011; Nielsen and El-Sohemy, 2012; Mavroidopoulou et al., 2015; Mählmann et al., 2016; Simonstein and Mashiach-Eizenberg, 2016; Horrow et al., 2019). However, in 43% of the studies a clear position toward the use of these tests is missing, hence their use in clinical practice remains controversial (Henneman et al., 2006, 2013; Morren et al., 2007; Hahn et al., 2010; Bombard et al., 2013; Haga et al., 2013; Nicholls et al., 2013; Vermeulen et al., 2014; Kaphingst et al., 2015; Miyamoto et al., 2016; Metcalfe et al., 2018; Rebitschek et al., 2019). Moreover, in contrast with the spread of DTC-GTs, the importance of pre- and post-test genetic counseling was underlined (Morin, 2009; Mai et al., 2011; Almeling, 2014; Mavroidopoulou et al., 2015).

In several studies, participants reported that genetic tests would be appropriate only if used for the diagnosis of treatable or preventable diseases (Morren et al., 2007; Vermeulen et al., 2014; Graves et al., 2015; Metcalfe et al., 2018). Furthermore, according to the study of Graves et al. (2015) the disease severity was also reported as an important parameter in deciding which test results are to be known (Covolo et al., 2015).

### Educational Needs

Twenty percent (*n* = 11) of the studies endorsed the issue of citizens' education (Table 2; Skirton et al., 2006; Morin, 2009; Hahn et al., 2010; Lemke et al., 2010; Dijkstra et al., 2012; East et al., 2012; Bombard et al., 2013; Etchegary et al., 2015; Kaphingst et al., 2015; Fournier and Poulain, 2018; Rebitschek et al., 2019). Most of them (82%, *n* = 9) highlighted a clear educational need to be fulfilled (Skirton et al., 2006; Morin, 2009; Hahn et al., 2010; Lemke et al., 2010; East et al., 2012; Bombard et al., 2013; Etchegary et al., 2015; Fournier and Poulain, 2018; Rebitschek et al., 2019), showing however a lack of proactivity in information seeking (Dijkstra et al., 2012). General population's education should focus, according to study participants, on the following main topics: genetic/genomics research (Hahn et al., 2010; Lemke et al., 2010; Dijkstra et al., 2012; Etchegary et al., 2015), disease etiology and susceptibility (Hahn et al., 2010; Kaphingst et al., 2015; Rebitschek et al., 2019), nutrigenomics (Morin, 2009), and genetic and omics tests (Fournier and Poulain, 2018; Rebitschek et al., 2019).

Furthermore, the importance of informed consent process as an educational moment (Kaphingst et al., 2012) and the priority and usefulness to pay particular attention to individuals at high risk for specific diseases, “isolated groups” (e.g., low income, ethnic minorities), youth and students were underlined (Lemke et al., 2010).

Moreover, during focus groups described by Lemke et al. several information strategies were suggested by participants, such as evening TV news, internet, and focus groups (Lemke et al., 2010).

Lastly, 4 studies (8%) investigated methods to increase citizens' literacy, such as Science Cafés with genomics experts (Ahmed et al., 2017), education courses on specific genetic and ethic topics (Gleason et al., 2010), short courses held by experts (East et al., 2012), and an institutional web information portal (Gleason et al., 2010). The comparison of described experiences doesn't allow to establish the most effective method to improve general population's literacy, due to the lack of quantitative analysis and the heterogeneity of initiatives and topics dealt with.

## Discussion

This review aimed to describe current literature about citizens' knowledge, attitudes, and educational needs in the field of omics sciences. We performed a systematic literature search and we identified studies published from 2006 onward, few years later than the completion of HGP in 2003 (International Human Genome Sequencing Consortium, 2004). Most of them were conducted in USA (Fomous et al., 2006; Goddard et al., 2007, 2009; Gleason et al., 2010; Hahn et al., 2010; Lemke et al., 2010; East et al., 2012; Kaphingst et al., 2012, 2015, 2016; Kolor et al., 2012; Haga et al., 2013; Almeling, 2014; Borzekowski et al., 2014; Waters et al., 2014, 2016, 2017; Abrams et al., 2015; Dodson et al., 2015; Graves et al., 2015; Schmidlen et al., 2016; Ahmed et al., 2017; Krakow et al., 2017; Horrow et al., 2019) and fewer in European Union (Henneman et al., 2006, 2013; Calsbeek et al., 2007; Morren et al., 2007; Mai et al., 2011; Smerecnik et al., 2011; Dijkstra et al., 2012; Vermeulen et al., 2014; Mavroidopoulou et al., 2015; McCormack et al., 2016; Fournier and Poulain, 2018; Rebitschek et al., 2019). The main promoters of identified initiatives were universities (Fomous et al., 2006; Henneman et al., 2006; Skirton et al., 2006; Morren et al., 2007; Morin, 2009; Stewart-Knox et al., 2009; Hahn et al., 2010; Mai et al., 2011; Smerecnik et al., 2011; Nielsen and El-Sohemy, 2012; Bombard et al., 2013; Haga et al., 2013; Nicholls et al., 2013; Almeling, 2014; Borzekowski et al., 2014; Vermeulen et al., 2014; Waters et al., 2014, 2016, 2017; Dodson et al., 2015; Etchegary et al., 2015; Graves et al., 2015; Kaphingst et al., 2015, 2016; Mavroidopoulou et al., 2015; Mählmann et al., 2016; Miyamoto et al., 2016; Schmidlen et al., 2016; Simonstein and Mashiach-Eizenberg, 2016; Fournier and Poulain, 2018; Metcalfe et al., 2018), enlightening the high interest of the scientific community for genomics and omics science implications for citizens and patients.

Main tools used to investigate citizens' attitudes, knowledge, and educational needs were questionnaires or surveys (Henneman et al., 2006, 2013; Calsbeek et al., 2007; Goddard et al., 2007, 2009; Morren et al., 2007; Ishiyama et al., 2008; Makeeva et al., 2009; Molster et al., 2009; Stewart-Knox et al., 2009; Sturgis et al., 2010; Mai et al., 2011; Smerecnik et al., 2011; Dijkstra et al., 2012; East et al., 2012; Kaphingst et al., 2012, 2015, 2016; Kolor et al., 2012; Nielsen and El-Sohemy, 2012; Haga et al., 2013; Almeling, 2014; Borzekowski et al., 2014; Vermeulen et al., 2014; Waters et al., 2014, 2016; Abrams et al., 2015; Dodson et al., 2015; Etchegary et al., 2015; Graves et al., 2015; Mavroidopoulou et al., 2015; Mählmann et al., 2016; Miyamoto et al., 2016; Schmidlen et al., 2016; Simonstein and Mashiach-Eizenberg, 2016; Krakow et al., 2017; Horrow et al., 2019; Jones et al., 2019; Pereira et al., 2019; Middleton et al., 2020) and, less frequently, residential events (Skirton et al., 2006; Morin, 2009; Hahn et al., 2010; Lemke et al., 2010; Bombard et al., 2013; Nicholls et al., 2013; Etchegary et al., 2015; McCormack et al., 2016; Ahmed et al., 2017; Waters et al., 2017; Fournier and Poulain, 2018; Metcalfe et al., 2018; Jones et al., 2019; Rebitschek et al., 2019). Just in few cases more innovative methods, such as a movie or a short video (Sturgis et al., 2010; Mählmann et al., 2016) or a web portal regarding genomics and its implications (Fomous et al., 2006), were adopted.

The results of the present systematic review show that citizens' knowledge on omics is generally poor or very poor (Skirton et al., 2006; Calsbeek et al., 2007; Goddard et al., 2007, 2009; Morren et al., 2007; Ishiyama et al., 2008; Molster et al., 2009; Morin, 2009; Gleason et al., 2010; Hahn et al., 2010; Lemke et al., 2010; Smerecnik et al., 2011; Dijkstra et al., 2012; East et al., 2012; Nielsen and El-Sohemy, 2012; Almeling, 2014; Borzekowski et al., 2014; Abrams et al., 2015; Etchegary et al., 2015; Mavroidopoulou et al., 2015; Mählmann et al., 2016; Simonstein and Mashiach-Eizenberg, 2016; Ahmed et al., 2017; Waters et al., 2017; Metcalfe et al., 2018; Jones et al., 2019). Thus, a clear need for improving citizens' literacy is evident, even to avoid risks deriving from inappropriate use of omics technologies (Ricciardi and Boccia, 2017; Boccia et al., 2019). The long time period covered by our systematic review, starting with the beginning of the omics era interestingly shows that over some 15 years only one study from 2019 of Horrow et al. (2019) reports a high level of awareness and knowledge about genomics. As the participants in this study took part in a genome sequencing project, this result is not surprising and points out that an adequate information associated with active citizen involvement improves not only knowledge but also perception and attitudes on omics sciences and, in particular, about genetic/omics tests.

Moreover, several studies investigated which sociodemographic factors can correlate with citizens' knowledge level and literacy. In particular, a positive association was shown with education (Kaphingst et al., 2012; Abrams et al., 2015) and younger age (Mai et al., 2011; Kaphingst et al., 2016). Furthermore, a lower knowledge level was shown in individuals residing in suburban and extraurban areas, compared with individuals living in cities (Mai et al., 2011).

Main sources of information resulted to be TV, magazines, newspapers, and internet, rather than healthcare professionals. This highlights the need for increasing literacy, not only for citizens, who should be better informed about where and from whom to seek trusted information, but for healthcare professionals too (Ricciardi and Boccia, 2017), in order to allow them interact with patients and give them satisfactory explanations (Boccia et al., 2017).

The present systematic review provides relevant information about citizens' perceptions regarding benefits and risks related to the use of genomics or omics sciences in the medical field too. In particular, most of the studies showed that participants perceived benefits and usefulness from the use of genomic and omics science in medicine (Skirton et al., 2006; Morren et al., 2007; Makeeva et al., 2009; Morin, 2009; Stewart-Knox et al., 2009; Hahn et al., 2010; Lemke et al., 2010; Nielsen and El-Sohemy, 2012; Bombard et al., 2013; Nicholls et al., 2013; Almeling, 2014; Mählmann et al., 2016; McCormack et al., 2016; Miyamoto et al., 2016; Simonstein and Mashiach-Eizenberg, 2016; Metcalfe et al., 2018). In detail, perceived benefits concentrated in the fields of prevention and treatment (Morren et al., 2007; Morin, 2009; Hahn et al., 2010; Nielsen and El-Sohemy, 2012; Bombard et al., 2013; Nicholls et al., 2013; Miyamoto et al., 2016), and in general were related to a potential improvement in life expectancy (Henneman et al., 2006; Makeeva et al., 2009). Instead, the main concerns of citizens relate to the privacy and use, sharing and access to data (Stewart-Knox et al., 2009; Lemke et al., 2010; Nicholls et al., 2013; Etchegary et al., 2015; Mavroidopoulou et al., 2015; Mählmann et al., 2016; Miyamoto et al., 2016; Metcalfe et al., 2018; Jones et al., 2019; Middleton et al., 2020), job discriminations (Henneman et al., 2006; Makeeva et al., 2009; Lemke et al., 2010; Almeling, 2014; Metcalfe et al., 2018), and the insurance issues (Calsbeek et al., 2007; Morren et al., 2007; Lemke et al., 2010; Haga et al., 2013; Henneman et al., 2013; Miyamoto et al., 2016; Middleton et al., 2020). Other concerns are related to potential consequences of genetic information on working and professional life (Morren et al., 2007; Haga et al., 2013; Miyamoto et al., 2016), costs (Bombard et al., 2013; Nicholls et al., 2013; Metcalfe et al., 2018), access to tests and feasibility of the introduction of genetic tests into clinical practice (Bombard et al., 2013). Few studies have also reported psychological distress in citizens potentially caused by genetic test results (Skirton et al., 2006; Mählmann et al., 2016).

Overall, a positive attitude toward genetic or omics tests was described (Skirton et al., 2006; Calsbeek et al., 2007; Makeeva et al., 2009; Morin, 2009; Stewart-Knox et al., 2009; Mai et al., 2011; Nielsen and El-Sohemy, 2012; Mavroidopoulou et al., 2015; Mählmann et al., 2016; Simonstein and Mashiach-Eizenberg, 2016; Horrow et al., 2019), even though with some concerns (Henneman et al., 2006, 2013; Morren et al., 2007; Hahn et al., 2010; Bombard et al., 2013; Haga et al., 2013; Nicholls et al., 2013; Vermeulen et al., 2014; Kaphingst et al., 2015; Miyamoto et al., 2016; Metcalfe et al., 2018; Rebitschek et al., 2019). Furthermore, the important role of pre- and post-test genetic consultation was underlined (Morin, 2009; Mai et al., 2011; Almeling, 2014; Mavroidopoulou et al., 2015). Some studies highlighted that key points for appropriateness of genetic tests are curability and preventability of the disease they are addressed to Morren et al. (2007); Vermeulen et al. (2014); Graves et al. (2015); Metcalfe et al. (2018).

Moreover, the few studies investigating need or request for more education or information on genomics or omics sciences among the population highlighted a clear lack to be fulfilled (Skirton et al., 2006; Morin, 2009; Hahn et al., 2010; Lemke et al., 2010; East et al., 2012; Bombard et al., 2013; Etchegary et al., 2015; Fournier and Poulain, 2018; Rebitschek et al., 2019). The main identified topics to focus for citizens' education were genetic/genomic research (Hahn et al., 2010; Lemke et al., 2010; Dijkstra et al., 2012; Etchegary et al., 2015), disease etiology and susceptibility (Hahn et al., 2010; Kaphingst et al., 2015; Rebitschek et al., 2019), nutrigenomics (Morin, 2009), and genetic and omics tests (Fournier and Poulain, 2018; Rebitschek et al., 2019).

Our results show also a set of potential information strategies, such as TV news, internet, and focus groups (Lemke et al., 2010), Science Cafés with genomic experts (Ahmed et al., 2017), education courses on specific genetic and ethic topics (Gleason et al., 2010), short courses held by experts (East et al., 2012), and an institutional web information portal (Gleason et al., 2010). However, unfortunately it was not possible to establish quantitatively the most effective method to improve citizens' literacy in omics sciences field.

Notably, we found no study focusing on omics sciences other than genomics and genetics or genetic tests. This could be due to genomic applications being more widespread than other omics technologies, which are probably still less known and more difficult to understand by the general population. Based on this knowledge gap, the planning of education programs for citizens should pay more attention to these issues and provide clear and accurate definitions of the different omics sciences and related tests. Indeed, an important aspect to take into consideration in the information/training process aimed at the general population is also the terminological consistency to be used and disseminated (Martin et al., 2020). Indeed, this is a key aspect for citizen empowerment, since the terminological inappropriateness or variety of scientific definitions could cause great confusion among citizens, especially in a field as complex as that of the omics sciences.

To our knowledge, no previous systematic review attempted to assess citizens' knowledge and attitudes in the wide field of omics sciences. We included also studies related to the field of nutrigenomics, genomics research, and biobanking. Furthermore, to date we do not yet have adequate information on the educational needs of the general population in these fields.

However, our study has several limitations too. In particular, due to the high heterogeneity among studies, it was not possible to summarize quantitatively investigated issues, even though we believe we appropriately summarize here our findings qualitatively. In addition, we included studies conducted on a limited number of participants which therefore may be not representative of the general population. Another limitation of the study is represented by the heterogeneity of the “citizens” studied in the papers like patients suffering from different diseases, participants in sequencing (biobank) projects, users of DTC-GTs etc. The different “citizens” studied obviously have different knowledge levels and training needs that must be taken into account in planning a training strategy for the general population. Also, there may be an insufficient understanding by the general population of “omics sciences” as merely “genetic.” This, however, seems to be kind of a general perception, also by professionals. The results of the present systematic review show that citizens' knowledge on omics is generally poor or very poor (Morin, 2009; Hahn et al., 2010; Lemke et al., 2010; Dijkstra et al., 2012; Metcalfe et al., 2018) though some papers show moderate knowledge of citizens on basic genetics and genetic testing (Mai et al., 2011; Haga et al., 2013; Schmidlen et al., 2016; Waters et al., 2016; Krakow et al., 2017) and the interest of the population on these issues is emerging (Skirton et al., 2006; Morin, 2009; Hahn et al., 2010; Lemke et al., 2010; Dijkstra et al., 2012; East et al., 2012; Bombard et al., 2013; Etchegary et al., 2015; Kaphingst et al., 2015; Fournier and Poulain, 2018; Rebitschek et al., 2019). These data, however, underline an important educational gap to be bridged in order to make all stakeholders (health professionals, decision makers, citizens, etc.) understand what omics sciences are and what their potential is for public health.

Additionally, the studies included in our systematic review were conducted in North America, the EU, the United Kingdom, Switzerland, Russia, Australia, Japan and Israel, and three studies were conducted “in various countries.” Therefore, our results are clearly biased toward Western culture and may not be representative of the world population. Due to the high heterogeneity among studies, it was not possible to give a clear differentiation of the range of application of our findings for the different world parts and cultures and further research will be needed aimed at defining the educational needs of citizens in the various countries. However, the aim of our study was to highlight current knowledge on citizens' literacy, attitudes, and educational needs on omics sciences, underlining the need for strengthening public engagement on this topic.

Despite the limitations described, our study provides important indications for fostering research and innovation in the field of omics sciences. In particular, it allows us to confirm citizens' literacy but also the capacity building of healthcare professionals in omics sciences field as a priority for action for health systems around the world. Healthcare professionals should be the main actors in the information process of citizens, especially in complex fields such as that of the omics sciences. The results of our review show that main sources of information for citizens resulted to be TV, magazines, newspapers, and internet. Therefore, an adequate training of health professionals is necessary not only to ensure the correct application of omics sciences in clinical practice, but also for correct information for end users and, therefore, for patients and citizens.

Furthermore, our systematic review identified the main elements to be considered for the development and implementation of new training strategies for citizens in the field of omics sciences. Specifically, based on the evidence collected (Tables 1–4), a training activity on omics sciences aimed at citizens must take into account the following items:

1. *Target population to be trained*. Training can be aimed at the general population or at specific population groups such as young or old people, students, individuals with specific diseases (e.g., patients with multifactorial diseases, chronic diseases, rare diseases, etc.), populations at greater risk for a given condition than the general population, workers, etc.
2. *Topics on which to train citizens*. Main topics on which to train citizens emerge from our study as the following: scientific basis of genetics, disease etiology and genetic susceptibility, genetic and non-genetic risk factors (environmental, lifestyle, etc.) on the risk of disease; possibilities, implications and future developments of genomics/omics research; genetic and omics tests with particular focus on DTC-GTs; importance of pre- and post-test genetic counseling; nutrigenomics and correlated tests; “genomic medicine,” “personalized medicine,” personalized approaches in the prevention of diseases; role of the omics sciences in specific fields (e.g., oncology, aging, cardiovascular, forensics); ethical implications associated with genetic/omics research and the use of genetic/omics tests, etc.
3. *Tools with which to train the population*. Our results show also a set of potential information strategies, such as TV news, internet, focus groups, Science Cafés with genomics experts, education courses on specific topics, short courses held by experts, and institutional web information portal.
4. *Professionals to be involved in the training of the population*. This aspect was not investigated in our review however our study reveals that the main source of information for citizens are TV, magazines, newspapers, and internet. These data associated with the low level of citizens' knowledge in the omics sciences field further underlines the central role that health professionals must have in the education of citizens.

The general understanding of omics and its benefits for medicine, particularly in the context of personalized medicine, still appears to be quite limited despite important scientific advances in this field and the broad marketing of DTC tests. However, considering the broader context of “big” health data, understanding the omics issues is even more complex and for the future implementation of omics sciences in healthcare systems, educating of both citizens and health professionals will be essential. Indeed, not all “experts” could fully grasp the potential, challenges and issues at stake if not properly trained. Healthcare is increasingly data-driven, including omics. Therefore, health professionals will need to be properly trained in order to enable to the patients more informed health choices and ensure greater quality of healthcare (Fiske et al., 2019).

Therefore, omics-based knowledge will be of utmost importance for every healthcare practitioner, regardless of the field of practice, as well as for citizens. Education will be crucial for the effective and successful implementation of omics sciences and it will have to evolve along with the changing scientific landscapes.

### Conclusions

Progress in genomics has clear and crucial implications for public health. The current scientific context suggests a rapid transition from conventional to personalized medicine. Therefore, a strategic line to define the promotion and governance of omics-related innovation is necessary.

In order to achieve this change, the involvement of all stakeholders such as healthcare professionals, leaders, decision and policy makers, and citizens, will be necessary. In addition, the progress of the omics sciences is linked to the need to develop a solid literacy of both healthcare professionals and citizens. For this reason, effective tools of knowledge on the omics sciences field, especially for citizens, will have to be identified and implemented. Hence, further research should be fostered in the future to allow the identification of appropriate and effective methods for the design and implementation of large-scale interventions aimed at improving citizens' literacy and engagement in the rapidly changing field of omics sciences.

## Author Contributions

SB and GEC conceived the study and MS and AT participated in its design. GEC, MS, and AT identified the studies through a search of MEDLINE, ISI Web of Science, and Embase online databases and performed the data extraction from the papers. SB and GEC supervised MS and AT. GEC, MS, and AT critically discussed and interpreted the results of the review. GEC and MS contributed equally to the drafting of the paper. SB critically reviewed this manuscript. All the authors approved the final version.

## Conflict of Interest

The authors declare that the research was conducted in the absence of any commercial or financial relationships that could be construed as a potential conflict of interest.
